# The Baron's complaint

**DOI:** 10.1186/2041-2223-2-18

**Published:** 2011-08-18

**Authors:** Mark A Jobling

**Affiliations:** 1 Department of Genetics, University of Leicester, University Road, Leicester LE1 7RH, UK

## 

Puzzlingly for those who actually lived through the decade, a 1980s revival has been underway for a good while. We've seen the renaissance of power dressing, leg warmers, big hair and the shoulder pad; Heaven 17 and the Human League have been on tour again.

There's fondness for these elements of 1980s life, cheesy though they may have been. The politicians of the time tended to be much more polarising. Two such figures, one deceased and one retired, made the news again recently. On US Independence Day, a new statue was unveiled outside the American Embassy in London. Ten feet tall, and cast in a chocolaty bronze, Ronald Reagan now smiles benignly down on passersby. One week previously, an iconic handbag belonging to Margaret Thatcher was sold for £25,000 at a charity auction.

Apart from being a mutual admiration society, these old transatlantic allies were united by a physical condition: They both suffered from something called Dupuytren's contracture. 'Whose what?', you are probably asking yourself. The overbearing and authoritarian Baron Guillaume Dupuytren was a French physician, and Napoleon Bonaparte's doctor. He described this thickening in the palm of the hand, and the progressive and permanent contraction of the fingers [1], in a lecture in 1831, and gave his name to the condition despite not being the first to report it. The contracture severely limits hand function; it can be corrected by surgery, but may recur, and can eventually require amputations.

Dupuytren's disease is more prevalent among men than women, and appears rather late in life: its average age of onset is 60 years. For a geneticist, the interest comes from its reported heritability: it possesses an Online Mendelian Inheritance in Man database number [OMIM:126900] and is described as a dominant trait with incomplete penetrance as well as the commonest heritable disorder of connective tissue. One linkage study in a five-generation Swedish pedigree [2] fits this pattern and provides evidence for a locus on chromosome 16q. For the historian, however, the condition is intriguing because of its alleged population distribution and origins: It is widely regarded as a 'Viking disease'.

The condition was always known to be relatively common in northern Europe and rare in other continents, but the Viking connection was first suggested explicitly in a paper published in 1962 [3] in which the frequency was found to be highest in Danes. The author hypothesised that, if the condition arose within the 'Nordic racial group', then its distribution elsewhere might be explained on the basis of Viking migration.

In a nice example of an interdisciplinary study [4], David Elliot, a plastic surgeon, and Diana Whaley, a professor of early medieval studies, collaborated to identify and interpret, in the medieval Viking sagas, descriptions of conditions that might have been Dupuytren's. They discovered four accounts of miracles from Orkney and Iceland in which people with disabled hands were cured. For example, from the 12th-century *Longer Saga of Magnus*: 'There was a man called Sigurðr, from north in Shetland. He had a crippled hand such that all the fingers lay in the palm. He went to the holy shrine ... and there found healing, with fingers straight and supple for all his needs.'

My research group has been interested in Viking ancestry in Britain for a while, and I first encountered Dupuytren's in the flesh after addressing DNA donors in Knowsley, near Liverpool. The condition was not mentioned in my talk, but afterwards an elderly man asked a question. "Do you know anything about this?," he asked. He held up a hand with ring and little fingers tightly contracted into his palm. His brother and father, he said, were sufferers too. I asked if anyone else in the audience had the condition, and, to my surprise, about 10 of the 200 people there said they did. The frequency revealed by this straw poll seemed remarkably high, and I wondered if the excess Norse Viking ancestry indicated in this population by our own Y-chromosomal studies [5] was connected to this high incidence. There again, maybe it was ascertainment bias: someone develops Dupuytren's contracture, is told 'it's a Viking disease', gets interested in their own possible Viking heritage, and so enrols in our study and comes to my talk.

In fact, published figures for the incidence of Dupuytren's can be much higher than this. There is confusion due to variable diagnostic criteria; differences in participant age; environmental issues, including manual work, smoking and alcohol; and risk factors such as diabetes or epilepsy [6]. However, in most populations of northern European ancestry, the prevalence of Dupuytren's is more than 10%, and in a Norwegian sample of males between 70 and 79 years of age, the figure is a remarkable 35%.

Back to the genetics. Unfortunately, since the population surveys were undertaken by surgeons and physicians rather than geneticists, most paid no attention to family history or mode of inheritance. But if Dupuytren's is caused by dominant alleles, and if there is a Viking founder mutation, then surely it should be easy to find the gene and map its migrations along with those of the voyagers who carried it. Alas, if only things were so simple. A recent genome-wide association study [7] of Dutch, German and British cases found nine loci associated with susceptibility to Dupuytren's contracture, six of which involved the Wnt signalling pathway. All have quite modest effects (the largest odds ratio is 1.98), and there is no sign of the 16q locus suggested by the Swedish family study.

So the Baron's complaint is probably more complex than it appears. There is certainly a need for more family studies, and perhaps a proper look at Scandinavia. But, sadly, it seems possible that the intriguing tale of the 'Viking disease' might be a myth (like the horned helmet and the addiction to rape and pillage) that people just want to be true. As the sufferer Lady Thatcher once said, 'Nothing is more obstinate than a fashionable consensus'.
